# A Transcriptomic Study of the Effects of Tea Tree Essential Oil on the Pathogenicity of *Candida albicans*

**DOI:** 10.3390/jof12050354

**Published:** 2026-05-12

**Authors:** Yutao Zhou, Jiahao Xu, Chang Su, Weina Wu, Fengping Yi

**Affiliations:** Department of Perfume and Aroma Technology, Shanghai Institute of Technology, Shanghai 201418, China; 17382129375@163.com (Y.Z.); 15026655141@163.com (J.X.); suchang@sit.edu.cn (C.S.)

**Keywords:** tea tree oil, *Candida albicans*, pathogenicity of *C. albicans*, ferroptosis

## Abstract

*Candida albicans* is a common opportunistic pathogen. Long-term use of azole antifungals faces challenges like resistance, necessitating novel agents. Tea tree oil (TTO), a natural broad-spectrum antimicrobial, shows promise, but its molecular mechanisms, particularly concerning novel cell death pathways, require clarification. This study comprehensively evaluated the antifungal mechanism of TTO against *C. albicans* using transcriptomics. Antifungal susceptibility assays were conducted to assess the effects of TTO and its components (4-terpineol, terpenes, and γ-pinene) on the growth of *C. albicans* hyphae and biofilms. Fluorescent labeling and biochemical analysis were employed to detect ferroptosis markers. Transcriptomic results indicate that TTO induces 423 differentially expressed genes and systematically inhibits the development of *C. albicans* hyphae through mechanisms such as oxidative stress, iron homeostasis disruption, disruption of cell wall integrity, and interference with ergosterol metabolism. Notably, the significant enrichment of redox enzyme activity and iron ion binding functions, along with changes in the glutathione metabolic pathway, suggest that ferroptosis may be involved in this process. Subsequent studies revealed that the compound 4-pinene most effectively inhibits the pathogenicity of *C. albicans* by suppressing its adhesion, hyphae formation, and biofilm formation, whereas terpinene induces the accumulation of reactive oxygen species (ROS) and increases lipid peroxidation in *C. albicans*; furthermore, following treatment with an iron-mediated apoptosis inhibitor, terpinene enhances the viability of the treated *C. albicans* cells.

## 1. Introduction

*Candida albicans*, as a commensal fungus residing on human mucosal surfaces, is also a clinically significant opportunistic pathogen commonly found in the oral cavity, upper respiratory tract, gastrointestinal tract, and urogenital tract [1]. Under immunologically stable conditions, it remains harmless. However, when host immunity is compromised or microbial dysbiosis occurs, it can activate pathogenicity through morphological transformation (yeast-to-hyphal transition). This biphasic shift not only enhances adhesion to host epithelial cells but also triggers pro-inflammatory responses and forms three-dimensional biofilm structures [2]. Research indicates that the biofilm extracellular matrix is a complex structure composed not only of β-glucan, mannan, and extracellular enzymes but also of other key constituents such as extracellular DNA, lipids, and proteins. This matrix forms a physical barrier that significantly increases *C. albicans*’ resistance to fluconazole [3].

Although azole drugs remain the primary choice for antifungal therapy, their prolonged use can exacerbate drug resistance, hormonal disruption, and hepatotoxicity [4]. Therefore, the development of novel, eco-friendly, and low-toxicity antimicrobial agents has become a research priority. Tea tree oil, a highly concentrated extract from the leaves of the Australian tea tree, is regarded as a representative natural antimicrobial agent due to its skin-friendly properties and broad-spectrum antimicrobial activity [5]. The antimicrobial and antifungal activities of TTO and its major component, 4-terpenol, have been reported to be effective against a wide range of microorganisms, including bacteria, yeasts, and filamentous fungi [6,7]. Compared to other essential oils, TTO exhibits more pronounced antifungal potential, with its antimicrobial activity stemming from the synergistic effects of its complex terpenoid compounds [8]. TTO is not only used as a topical antimicrobial agent for the treatment of acne, but also serves as a natural biocide for controlling postharvest diseases in fruits and vegetables [5,9]. However, research on the antifungal effects of TTO is still relatively limited, especially regarding whether it exerts its effects through novel programmed cell death pathways [10].

Ferroptosis is a regulated, iron-dependent form of cell death distinct from apoptosis, necroptosis, and autophagy in both morphology and mechanism [11]. A key feature is the iron-catalyzed accumulation of lipid peroxides generated from reactive oxygen species, which ultimately compromises membrane integrity [11]. This process can be specifically blocked by ferroptosis inhibitors such as ferrostatin-1 and liproxstatin-1, which function as radical-trapping antioxidants or indirectly support peroxidase 4(GPX4) activity [12]. Although initially described in mammalian systems, recent evidence suggests that ferroptosis also occurs in microbes, including the pathogenic fungus *C. albicans* [13,14]. Given that *C. albicans* infections often involve iron-rich environments (e.g., mucosal surfaces, blood) and that the fungus is exposed to host-derived oxidative stress, the ferroptosis pathway may represent an important determinant of fungal survival or death during infection [10].

## 2. Materials and Methods

### 2.1. Reagents

Tea tree oil was purchased from Shanghai YuKuai Biotechnology Co., Ltd. (Shanghai, China). The standard components 4-terpineol (4-SYC), γ-terpinene (γ-SYX), and terpinene (TPYX) (purity ≥ 98%) were obtained from Shanghai Aladdin Biochemical Technology Co., Ltd. (Shanghai, China). *Candida albicans* (strain CMCC(F)98001) was acquired from a national microbial culture collection center in China. Dimethyl sulfoxide (DMSO), the ferroptosis inhibitor Ferrostatin-1 (Fer-1), glutathione (GSH), N-acetylcysteine (NAC), and the fluorescent probe 2,7-dichlorodihydrofluorescein diacetate (DCFH-DA) were all purchased from Shanghai Titan Technology Co., Ltd. (Shanghai, China). Malondialdehyde (MDA) assay kit and MTT cell proliferation/toxicity assay kit from Biyuntian Biotechnology Co., Ltd. (Shanghai, China).

### 2.2. C. albicans Culture

*C. albicans* was cultured in Yeast Extract-Peptone-Dextrose (YPD) medium at 28 °C with shaking for 18 h to reach the logarithmic growth phase, achieving a suspension concentration of approximately 1 × 10^7^ CFU/mL. The cells were collected by centrifugation, the supernatant was removed, and the pellet was resuspended and cultured in RPMI 1640 medium supplemented with 10% fetal bovine serum (FBS) at 37 °C to induce the hyphal form.

### 2.3. GC-MS Analysis

Volatile components of the essential oil were analyzed using a gas chromatography-mass spectrometry system (7890C-5973N, Agilent Technologies, Santa Clara, CA, USA). Separation was performed on a polar INNOWAX quartz capillary column (60 mm × 0.25 mm, 0.25 μm film thickness) with a flame ionization detector (FID). The injector temperature was set at 230 °C. A sample volume of 0.2 μL was injected with a split ratio of 30:1, using helium as the carrier gas. The oven temperature program was as follows: initial temperature 40 °C, increased to 120 °C at 5 °C/min, held for 2 min, then increased to 200 °C at 10 °C/min and held for 10 min.

### 2.4. Antifungal Activity Study

#### 2.4.1. Determination of Growth Curves

Transfer 500 μL of *C. albicans* culture in the logarithmic growth phase to 100 mL of YPD medium. Add tea TTO to achieve concentrations of 0 μL/mL, 0.5 μL/mL, 1 μL/mL, and 2 μL/mL, respectively. Incubate the cultures on a shaking incubator at 28 °C and 180 rpm. Every hour, the absorbance at 600 nm (OD_600_) of the culture medium was measured using a UV-6000 UV-visible spectrophotometer (Shanghai Metash Instruments CO., LTD., Shanghai, China) to monitor the growth of *C. albicans* at different concentrations of TTO (observations were made for a total of 14 h, with the final measurement taken at 24 h).

#### 2.4.2. Comparative Antifungal Activity

*C. albicans* was continuously cultured in YPD medium at 120 rpm for 18 h until the logarithmic growth phase, reaching a concentration of approximately 1 × 10^7^ CFU/mL. A specific volume of TTO, 4SYC, TPYX, and γSYX was added to centrifuge tubes containing 5 mL of the fungal culture medium, achieving final concentrations of 0, 1, 2, 4, and 8 µL/mL, respectively. After diluting the fungal suspension to 10^−4^, 10^−5^, and 10^−6^, 100 μL from each dilution was plated onto Bengal red agar medium. The plates were incubated at 28 °C for 36 h, and the colonies were counted manually.

#### 2.4.3. Microstructural Analysis

After culturing the two forms of *C. albicans* suspensions according to the method described in Section 2.2. TTO, 4-SYC, TPYX, and γ-SYX were added to the suspensions of each form at a concentration of 2 μL/mL. At the same time, two control suspensions containing no test compounds (0 μL/mL) were prepared. After incubating at 28 °C for 12 h, the supernatant was removed by centrifugation. Subsequently, the samples were fixed in 2.5% glutaraldehyde at 4 °C for 24 h. The samples were dehydrated in a gradient of 50% to 100% ethanol (in 10% increments). They were then treated with equal volumes of isovaleric acid and anhydrous ethanol, followed by treatment with 100% isovaleric acid. The samples were air-dried for 24 h and gold-sputtered for 1 min prior to observation under a scanning electron microscope (SEM). Images of the yeast form of *C. albicans* were captured at magnifications of 300 KX and 1000 KX, respectively, while images of the hyphal form of *C. albicans* were captured at a magnification of 300 KX.

### 2.5. Transcriptomics

Investigating the Effects of TTO on the Hyphal Formation Process of *C. albicans* via Transcriptomic Analysis. *C. albicans* was cultured in YPD medium at 28 °C under shaking conditions for 18 h until the logarithmic growth phase was reached, with a fungal suspension concentration of approximately 1 × 10^7^ CFU/mL; the fungal suspension was centrifuged to remove the supernatant. The JS group (JS1, JS2, and JS3) was cultured in RPMI 1640 medium supplemented with 10% fetal bovine serum at 37 °C for 6 h to form a mycelial state; the TTO group (TTO1,TTO2, and TTO3) was additionally treated with TTO at 1 μL/mL concentration for the same duration; The cells were then centrifuged at 8000 rpm for 10 min multiple times to collect the cells into “soybean-sized” pellets. After washing three times with Saline solution, the supernatant was aspirated, and the pellets were flash-frozen with dry ice before being sent to Sanshu Biotechnology for transcriptomic analysis.

Total RNA was extracted using a Trizol-based total RNA extraction kit (Sangon Biotech, Shanghai, China). RNA integrity was assessed by 1% agarose gel electrophoresis, and RNA quality was verified using an Agilent 2100 Bioanalyzer (Agilent, CA, USA), with all samples having an RNA Integrity Number (RIN) > 7.0. High-quality RNA was used to construct cDNA libraries, which were sequenced on an Illumina NovaSeq 6000 platform. Raw sequencing data quality was assessed using FastQC software (Fastp, 0.23.4; QualiMap, v.2.3). After filtering out low-quality reads, clean reads were aligned to the *C. albicans* SC5314 reference genome. Differentially expressed genes (DEGs) were identified using the R package edgeR (edgeR, 4.0.16; DESeq2, 1.42.0) with the thresholds of |fold change| > 2 and *p*-value < 0.05.

### 2.6. Study on the Ferroptosis Induced by TTO Against C. albicans

#### 2.6.1. Reactive Oxygen Species Within Cells

Intracellular Reactive oxygen species levels were measured using the fluorescent probe DCFH-DA according to the manufacturer’s instructions. Briefly, a *C. albicans* spore suspension (10^7^ CFU/mL) was centrifuged at 8000 rmp for 5 min and washed twice with 0.9% NaCl solution. The cells were then loaded with 10 μM DCFH-DA by incubation at 37 °C in the dark for 30 min. After washing, the cells were treated with 1 μL/mL TTO or its major components. ROS production was visualized and assessed using fluorescence microscopy (Olympus IX73, Shanghai Tonghao Photoelectric Technology Co., Ltd., Shanghai, China).

#### 2.6.2. Malondialdehyde (MDA)

The suspensions were treated with 1 μL/mL of TTO, 4SYC, TPYX, or γSYX for 6 h. After treatment, cells were harvested, and approximately 1 g (wet weight) of cell pellet was ground in liquid nitrogen. Nine volumes of PBS buffer were added to prepare a homogenate, which was then centrifuged at 12,000 rmp at 4 °C for 15 min. The supernatant was collected, and the MDA content was determined according to the instructions of the commercial assay kit.

#### 2.6.3. Iron Death Inhibitor

Cell suspensions were prepared in RPMI 1640 medium supplemented with 10% FBS. The suspensions were pretreated for 30 min with varying concentrations of the ferroptosis inhibitor Fer-1, GSH, or the ROS scavenger NAC. Subsequently, TTO or its components (4SYC, TPYX, and γSYX) were added at a concentration of 1 μL/mL. Cell viability was then assessed using an MTT assay kit.

### 2.7. Inhibit Hyphal Transformation and Biofilm Formation

#### 2.7.1. Biofilm Formation

*Candida albicans* suspension (100 μL) was inoculated into wells of a 96-well plate containing RPMI 1640 medium with 10% FBS to allow for biofilm formation. At specific time points during biofilm development (1.5, 6, and 24 h), the medium and non-adherent cells were aspirated. Fresh RPMI 1640 medium (100 μL) containing 1 μL/mL of TTO, 4SYC, TPYX, or γSYX was added to the respective wells. After 24 h incubation at 37 °C, the biofilm was gently washed three times with 100 μL PBS. The biofilm was then stained with 100 μL of 0.1% crystal violet for 15–20 min at room temperature. After staining, the wells were washed thoroughly with PBS until the wash solution became colorless. Bound dye was eluted with 100 μL of anhydrous ethanol for 10–15 min with shaking. The eluate was transferred to a new plate, and its absorbance was measured at 570 nm using a microplate reader.

#### 2.7.2. Adhesion Assay

A *Candida albicans* suspension (100 μL) was inoculated into wells of a 96-well plate. TTO or its components (4SYC, TPYX, γSYX) were added to the suspension at a concentration of 1 μL/mL, with an untreated cell suspension serving as the control. After incubation at 37 °C for 3 h, the liquid medium was removed, and the wells were washed three times with PBS to remove non-adherent cells. Adherent cells were stained with 0.1% crystal violet, and the dye was eluted and quantified as described in Section 2.7.1. The relative adhesion rate was calculated as: (OD_570_ of treated group/OD_570_ of control group) × 100%.

#### 2.7.3. Hyphal Formation Assay

The effects of TTO and its components on the morphological transition of *C. albicans* were investigated. Cells from an overnight YPD culture were collected, resuspended, and incubated at 37 °C for 6 h in RPMI 1640 medium with 10% FBS containing 1 μL/mL of TTO, 4SYC, TPYX, or γSYX. A control sample was incubated in the same medium without treatment. After incubation, 20 μL of the cell suspension was placed on a glass slide, air-dried, stained with crystal violet, and examined under a light microscope (1000× magnification) to observe yeast and hyphal cells.

### 2.8. Statistical Analysis

All experiments were repeated three times. Experimental data are expressed as mean ± standard deviation (SD). Origin software (Origin 2021, 9.80.200**)** was used to analyze the experimental results via one-way analysis of variance (ANOVA). When the F-value was significant (*p* < 0.05), Tukey’s test was employed to compare mean differences at a 5% significance level.

### 2.9. Data Availability

RNA-seq data are available in NCBI under BioProject accession number PRJNA10701.

## 3. Results

### 3.1. Antimicrobial Activity of TTO Against Candida albicans

#### 3.1.1. GC-MS Data Analysis

Table 1 presents the GC-MS analysis results for tea tree oil, with a total of 25 compounds detected. The table displays the top ten components identified in the GC-MS analysis of TTO, accounting for 88.88% of the total constituents. The GC-MS analysis indicates that monoterpenes are the dominant components in TTO, with terpinene-4-ol (26.54%), γ-terpinene (20.14%), and terpinene (17.26%) forming the core components, collectively accounting for 63.94%. These three major constituents were compared with TTO to investigate the antifungal effects and mechanisms of different components in TTO.

##### 3.1.2. The Effect of TTO on the Growth Curve of *Candida albicans*

**Figure 1:**
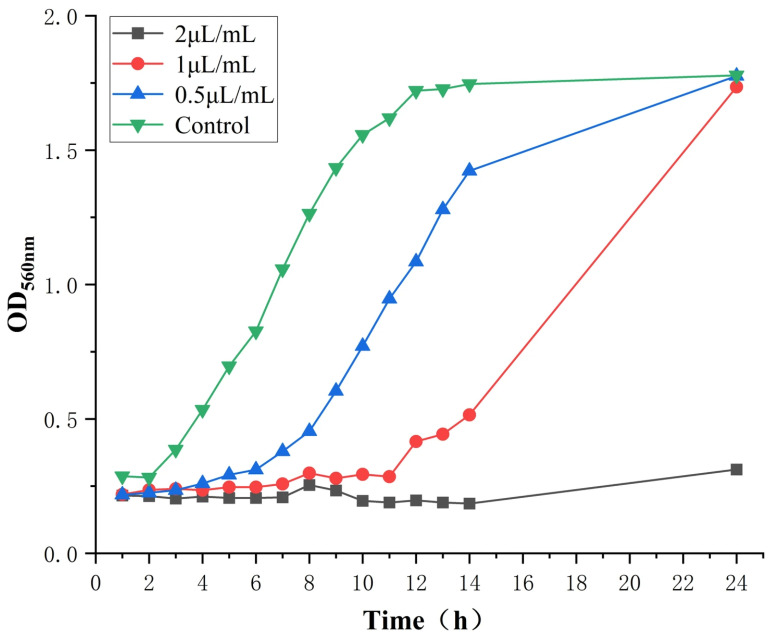
Experimental growth curve of *C. albicans* with TTO.

Based on the experimental results of the 24 h growth curve of *C. albicans* exposed to TTO, significant differences in fungal growth were observed across different concentration treatments (Figure 1). In the 2 μL/mL concentration group, the OD value remained stable between 0.20 and 0.25 throughout the 24 h observation period, showing no upward trend, indicating that the growth of *C. albicans* was completely inhibited; this concentration maintained a sustained antifungal effect for 24 h. In the 1 μL/mL group, growth was slow between 0 and 8 h; after 8 h, growth began to increase markedly, and by 12 h, the culture entered a rapid growth phase. At 24 h, the OD value reached 1.80, which was even slightly higher than the 1.70 observed in the blank control group (0 μL/mL), demonstrating a pattern of initial inhibition followed by delayed explosive growth. The 0.5 μL/mL group exhibited weak inhibitory effects, with growth only slightly delayed during the first 4 h; it subsequently entered the logarithmic growth phase rapidly, reaching an OD value of 1.85 at 24 h, which was essentially on par with the blank control group. The blank control group (0 μL/mL) exhibited a typical S-shaped growth curve, with the 0–4 h period serving as the adaptation phase, the 4–12 h period as the logarithmic growth phase, and the period after 12 h entering the stationary phase. The OD value at 24 h stabilized at around 1.70, indicating the reliability of the experimental system.

##### 3.1.3. Comparative Antifungal Activity

**Figure 2:**
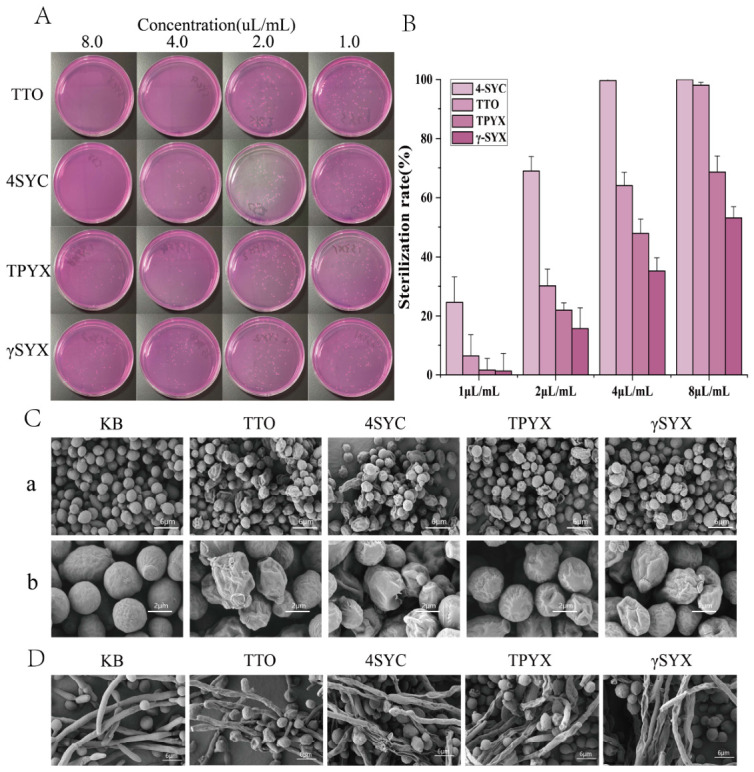
Antifungal activity of TTO and its three small-molecule compounds against *C. albicans*, along with post-treatment scanning electron micrographs. (**A**,**B**) Comparative evaluation of the fungicidal effects of TTO and its three major components against *C. albicans*. (**C**) SEM show the effects of TTO and its three main components on the ultrastructure of *C. albicans* yeast cells. (**a**) Overall view, scale bar = 6 μm. (**b**) Detail view, scale bar = 2 μm. (**D**) SEM images show the effects of TTO and its three main components on the ultrastructure of *C. albicans* hyphae, scale bar = 6 μm.

Figure 2A,B investigated the effect of different concentrations of TTO and its main components on the fungicidal rate against *Candida albicans*. As shown in Figure 2A, the number of *C. albicans* colonies gradually decreased with increasing concentrations of TTO and its components, indicating enhanced inhibitory effects. Among these, 4SYC exhibited the strongest inhibitory effect against *C. albicans*; at a concentration of 4.0 μL/mL, all colonies on the plate disappeared. TTO demonstrated the next best antifungal effect, with only two colonies remaining on the plate at a concentration of 8.0 μL/mL. This indicates that both 4SYC and TTO at their respective concentrations killed the vast majority of *C. albicans*. This result is better illustrated in Figure 2B. At a concentration of 4.0 μL/mL, the kill rates for the 4SYC, TTO, TPYX, and γSYX groups were 99.68%, 64.08%, 47.90%, and 35.28%, respectively. Results in Figure 2A,B indicate that the 4SYC group demonstrated the highest bactericidal efficacy, followed by the TTO, TPYX, and γSYX groups.

##### 3.1.4. Microstructural Analysis

The yeast form of *C. albicans* exhibits a single-celled oval structure that reproduces via budding. This yeast form is commonly observed in superficial infections (such as mucosal colonization) and during the early stages of biofilm formation. The integrity of its cell membrane is crucial for maintaining osmotic pressure and metabolic activity. Figure 2C shows a scanning electron micrograph of the cell membrane surface of *C. albicans* yeast cells after treatment with TTO and its primary small-molecule compounds. KB group refers to the blank control (untreated *C. albicans*). SEM observations reveal KB group cells exhibit typical oval shapes with smooth surfaces, distinct edges, and intact cell walls, showing no membrane rupture or leakage—indicating normal growth. TTO group cells display significant deformation (e.g., collapse, wrinkling), with some cells having ruptured and rough surfaces, indicating compromised membrane integrity. In the 4SYC group, cell edges were blurred, some cells collapsed, and surfaces exhibited depressions or holes, possibly indicating physical damage from small molecules penetrating the cell wall. In the TPYX group, cell morphology was distorted with irregular protrusions, but localized membrane dissolution occurred with minimal leakage of contents. In the γSYX group, cells showed slight deformation and increased surface roughness, yet the overall structure remained relatively intact.

The hyphal form of *C. albicans* is induced from the yeast form by the host microenvironment (e.g., serum, 37 °C, anaerobic conditions) and manifests as slender, multicellular filamentous structures; its ability to extend is directly related to the maturity of the biofilm, and inhibiting hyphal formation can effectively block its invasion of human tissues. Figure 2D shows scanning electron micrographs of the cell membrane surfaces of *C. albicans* hyphae after treatment with TTO and its major small-molecule compounds. As shown in the figure: hyphae in the KB group were slender, uniform, and smooth-surfaced, with clear branching structures; spores attached to the hyphal surface were regular in shape, with no rupture or deformation; In the TTO group, the hyphae were significantly deformed, with surface collapse and wrinkling; in the 4SYC group, the hyphae were shortened, with reduced branching, and the hyphal surface exhibited wrinkling or even rupture; although the hyphae in the TPYX and γSYX groups also showed some degree of indentation and wrinkling, the extent of damage was less severe than that in the 4SYC group.

### 3.2. Transcriptomics

**Figure 3:**
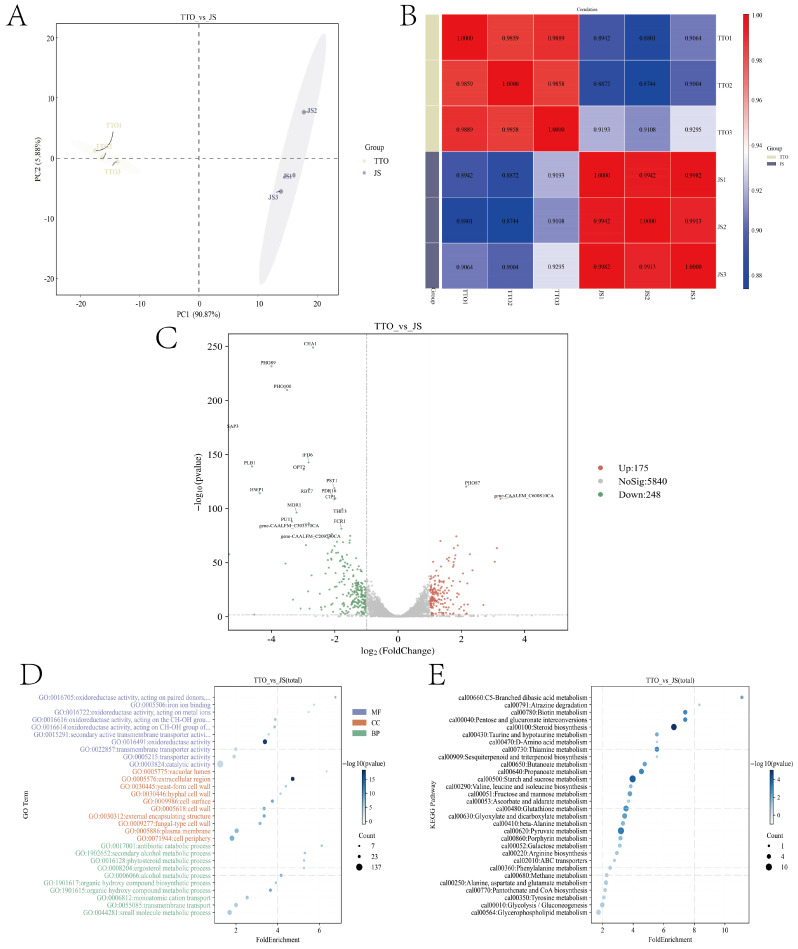
Transcriptome analysis of apoptosis in *C. albicans* hyphae under TTO stress. (**A**) Principal component analysis Plot. (**B**) Correlation coefficient plot. (**C**) Volcano plot of differentially expressed genes. (**D**) Gene Ontology (GO) enrichment plot. (**E**) Kyoto Encyclopedia of Genes and Genomes (KEGG) enrichment plot. Differential expression genes (DEGs) were selected using the R package edgeR or DESeq2 (1.42.0) with criteria of *p*-value < 0.05 and fold change > 2 or fold change < 0.5.

The treatment groups (TTO1, TTO2, and TTO3) and the control groups (JS1, JS2, and JS3) exhibited a clear trend of separation in the Principal component analysis plot (Figure 3A). The correlation coefficients between samples within the treatment groups were all higher than 0.98, and those within the control groups were all higher than 0.99, indicating good biological reproducibility within the groups and a high degree of consistency in the data (Figure 3B).

This study conducted a differential expression gene (DEG) analysis based on transcriptomic sequencing data. A total of 423 significantly differentially expressed genes were identified, including 175 up-regulated and 248 down-regulated genes (Figure 3C). As shown in the volcano plot, the transcriptome of *C. albicans* underwent relatively minor changes following TTO treatment. Among the significantly down-regulated genes, many virulence factor genes closely associated with the pathogenicity of *C. albicans* were markedly suppressed. For example, SAP3, which belongs to the secreted aspartic protease family, showed reduced expression, suggesting that TTO may reduce hyphal invasiveness by inhibiting protease activity; the expression of PST1 (involved in cell wall integrity) was also downregulated, which may affect cell wall structure and hyphal stability [15,16]. Additionally, downregulation was observed in the genes OPT2 (oligopeptide transporter) and IFD6 (aldehyde-ketone reductase family), which are associated with fungal metabolic adaptation and oxidative stress responses; these genes may be involved in the ferroptosis regulatory network induced by TTO [17,18]. Overall, TTO treatment regulates multiple genes associated with virulence, oxidative stress, and basal metabolism in a synergistic manner, providing a comprehensive molecular basis for elucidating its antifungal mechanism.

Gene Ontology (GO) functional enrichment analysis (Figure 3D) indicates that, at the molecular functional level, the most significantly enriched terms are primarily related to oxidoreductase activity, such as “Oxidoreductase activity acting on paired substrates” (GO:0016705, Fold Enrichment(FE):6.77), “Iron ion binding” (GO:0005506, FE:5.74), and “Oxidoreductase activity acting on metal ions” (GO:0016722, FE:5.48). The significant enrichment of these terms suggests, on the one hand, that the mycelial state is under severe oxidative stress following TTO treatment, and on the other hand, that iron metabolism is significantly disrupted. When considered in conjunction with the enrichment of iron ion binding functions, this further supports our previous hypothesis—that TTO may exert its antifungal effects through the ferroptosis pathway. Additionally, “redox activity acting on CH-OH groups” (GO:0016616, FE:3.88/GO:0016614, FE:3.85) was also significantly enriched, indicating that the pathway enrichment results are consistent. At the cellular compartment level, significantly enriched compartments include the vacuolar lumen (GO:0005775, FE:6.35), the extracellular space (GO:0005576, FE:4.71), as well as the yeast-type cell wall (GO:0030445, FE:4.39) and the hyphal-type cell wall (GO:0030446, FE:4.14). Notably, the hyphal-type cell wall was also observed as an enriched item. Regarding biological processes, the enriched terms span several metabolic pathways. Among these, the antibiotic catabolism process (GO:0017001, FE:6.11), the secondary alcohol metabolism process (GO:1902652, FE:5.30), and the ergosterol metabolism process (GO:0008204, FE:5.27) are particularly notable. Ergosterol is a key component of fungal cell membranes and a target of azole drugs. Furthermore, the enrichment of cation transport (GO:0006812, FE:2.53) and transmembrane transport (GO:0055085, FE:2.01) further indicates that TTO also disrupts ion homeostasis and nutrient uptake.

Kyoto Encyclopedia of Genes and Genomes (KEGG) pathway enrichment analysis (Figure 3E) revealed that differentially expressed genes were significantly enriched across multiple metabolic pathways, indicating that TTO treatment had a broad-ranging impact on the metabolic network of the mycelial state. The most significantly enriched pathway was C5-branched dicarboxylic acid metabolism (cal00660, FE:11.11). This pathway is closely associated with the degradation of branched-chain amino acids such as valine, leucine, and isoleucine. Its enrichment indicates that TTO treatment severely disrupted the amino acid metabolic homeostasis of *C. albicans*. Concurrently, the valine, leucine, and isoleucine biosynthesis pathway (cal00290, FE:3.85) also appeared on the enrichment list, further confirming that the branched-chain amino acid metabolic pathway has indeed been globally disrupted. The enrichment of the glutathione metabolism pathway (cal00480, FE:3.53) provides a significant mechanistic clue: glutathione is a central molecule in maintaining redox balance in eukaryotes and a key regulator of ferroptosis. Changes in this pathway align with the results from the previous GO analysis regarding redox enzyme activity, providing pathway-level evidence that TTO induces ferroptosis in *C. albicans*. Regarding energy metabolism, several central carbon metabolism pathways were significantly affected, including pyruvate metabolism (cal00620, FE:3.21), oxaloacetate and dicarboxylic acid metabolism (cal00630, FE:3.45), glycolysis/ gluconeogenesis (cal00010, FE:1.98), fructose and mannose metabolism (cal00051, FE:3.79), and starch and sucrose metabolism (cal00500, FE:3.97). The disruption of these pathways indicates that the energy supply system of the hyphal state is disrupted following TTO treatment, which is consistent with the phenotype of impaired hyphal extension. Enrichment was also observed in the sterol biosynthesis pathway (cal00100, FE:6.67). Sterols (primarily ergosterol) are key components of fungal cell membranes and classic targets for azole antifungal agents; changes in this pathway are corroborated by the results of GO analysis related to ergosterol metabolism, suggesting that TTO may affect hyphal cell membrane integrity and fluidity by interfering with sterol synthesis.

### 3.3. Study on the Ferroptosis Induced by TTO Against C. albicans

#### 3.3.1. Reactive Oxygen Species Scavenging

**Figure 4:**
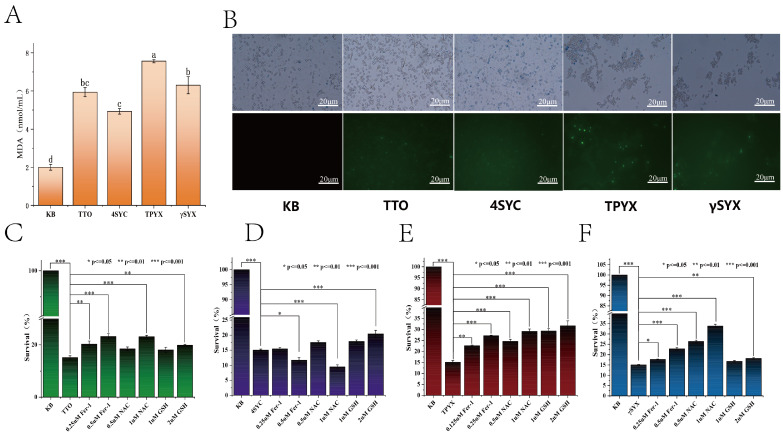
TTO and its three small-molecule derivatives induce iron-dependent cell death in *C. albicans*. (**A**) MDA content in *C. albicans* under 4 μL/mL TTO, 4SYC, TPYX, and γSYX. (**B**) Reactive Oxygen Species (ROS) bursts detected by fluorescence microscopy using the 2,7-dichlorodihydrofluorescein diacetate (DCHF-DA) probe under Fe^2+^ exposure. (**C**–**F**) Rescue of TTO (**C**), 4SYC (**D**), γSYX (**E**), and TPYX. (**F**)-induced iron-mediated death in *C. albicans* by Ferrostatin-1 (Fer-1), glutathione (GSH) and N-acetylcysteine (NAC). Note: Different lowercase letters in Figure A indicate significant differences (*p* < 0.05). The asterisks in C–F indicate statistically significant differences between groups: * *p* < 0.05, ** *p* < 0.01, *** *p* < 0.001.

The accumulation of Reactive Oxygen Species can induce apoptosis or even necrosis through cellular oxidative stress. The 2,7-dichlorodihydrofluorescein diacetate (DCHF-DA) staining assay demonstrates intracellular ROS accumulation. Generally, stronger green fluorescence indicates higher levels of ROS production within cells. As shown in Figure 4B, no green fluorescence was observed in the control group (KB), whereas all groups treated with TTO and its major components exhibited green fluorescence, with the TPYX and γSYX groups displaying more pronounced green fluorescence. This indicates that TTO and its major small-molecule compounds induced ROS accumulation in *C. albicans*, with TPYX and γSYX playing a primary role in inducing ROS accumulation.

#### 3.3.2. Lipid Peroxides

Malondialdehyde is one of the key products of lipid peroxidation. Following exposure to ROS, elevated levels of lipid peroxidation damage phospholipids, enzymes, nucleic acids, and biological membranes; MDA content reflects the extent of lipid peroxidation. As shown in Figure 4A, MDA levels in all four treatment groups exhibited a significant increase compared to the control group (KB), with the most pronounced increase observed in the TPYX group. The MDA levels in the TTO, 4SYC, γSYX, and TPYX groups were 5.93, 4.93, 7.57, and 6.3 mmol/mL, respectively. Therefore, the primary component in TTO influencing MDA levels is TPYX, followed by γSYX and 4SYC.

#### 3.3.3. Iron Death Inhibitor

The study investigated the inhibition of iron-dependent cell death in *C. albicans* by TTO and its major small-molecule compounds using three inhibitors: the Fenton reaction inhibitor Fer-1, the reactive oxygen species scavenger NAC, and the lipid peroxidation inhibitor GSH (Figure 4C–F). Based on MTT reduction assays, the percentage of *C. albicans* cell viability was calculated and compared with the untreated control. Figure 4C–F demonstrate that all three inhibitors increased *C. albicans* viability; After TPYX treatment, *C. albicans* viability was enhanced by all three inhibitors, with the 2μM GSH group showing the strongest effect, increasing viability by 100% relative to the blank group (Figure 4F). Following γSYX treatment, viability was primarily increased by Fer-1 and NAC, with the 1 μM NAC group exhibiting the strongest effect at 2.5 times that of the blank group (Figure 4E). 4SYC-treated *C. albicans* activity was enhanced only by GSH, with relatively poor efficacy (Figure 4D). Among these, the TPYX group showed the best effect. Compared to TPYX treatment alone, different concentrations of ferroptosis inhibitors significantly increased *C. albicans* cell viability. This experiment indicates that the most effective component for *C. albicans* ferroptosis in TTO is TPYX, followed by γSYX.

### 3.4. In Vitro Simulation Experiment

#### 3.4.1. Adhesiveness

**Figure 5:**
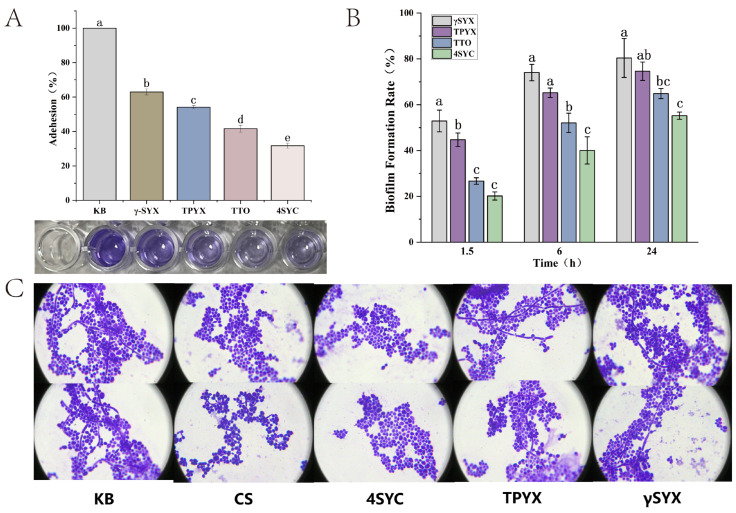
In vitro simulation experiments demonstrating the inhibition of *C. albicans* pathogenicity by TTO and its three small-molecule compounds. (**A**) Effects of TTO, 4SYC, TPYX, and γSYX on *C. albicans* biofilm adhesion. (**B**) Effects of TTO, 4SYC, TPYX, and γSYX on different stages of *C. albicans* biofilm formation. (**C**) Changes in *C. albicans* biofilm formation during mycelium-induced culture medium exposure to TTO, 4SYC, TPYX, and γSYX, visualized by crystal violet staining. Note: Different lowercase letters indicate significant differences (*p* < 0.05).

Figure 5A demonstrates the effects of TTO and its small-molecule compounds on *C. albicans* adhesion. The percentage of *C. albicans* adhesion was calculated by comparing the OD value of crystal violet at 570 nm with the untreated blank group. The adhesion rates for the γSYX group, TPYX group, TTO group, and 4SYC group were 62.97%, 54.15%, 41.62%, and 31.7%, respectively. At the same time, significance analysis indicated that there were significant differences in the ability of different compounds to inhibit the adhesion of *C. albicans*. With the lowest adhesion rate observed in the 4SYC group, followed by the TTO group. This indicates that 4SYC exhibits the strongest inhibitory effect on *C. albicans* adhesion. Furthermore, it suggests that 4SYC plays the primary role in inhibiting *C. albicans* adhesion within TTO, followed by TPYX and γSYX. Adhesion to epithelial cells marks the initial stage of *C. albicans* infection; inhibiting this adhesion completely blocks its ability to invade cells.

#### 3.4.2. Inhibit Biofilm Formation

Figure 5B illustrates the effects of TTO and its low-molecular-weight compounds on the formation of *C. albicans* biofilms. During the initial stage, biofilm formation rates for the γSYX, TPYX, TTO, and 4SYC groups were 52.92%, 44.71%, 26.64%, and 20.14%, respectively. This indicates that *C. albicans* biofilm formation was relatively highest in the γSYX group during the early adhesion stage, while TTO and 4SYC demonstrated superior inhibitory effects. During the development stage, biofilm formation rates were 74.05%, 65.24%, 52.08%, and 40.04% for the γSYX, TPYX, TTO, and 4SYC groups, respectively. At this stage, no significant difference was observed between the γSYX and TPYX groups (both *p* > 0.05), while TTO and 4SYC continued to exhibit inhibitory effects, with 4SYC demonstrating a more pronounced inhibitory effect. At the mature stage, biofilm formation rates for the γSYX, TPYX, TTO, and 4SYC groups were 80.40%, 74.62%, 64.88%, and 55.21%, respectively. This indicates that the γSYX group exhibited the highest biofilm formation at the mature stage, while 4SYC demonstrated the most effective inhibition of biofilm formation.

#### 3.4.3. Inhibit Mycelium Formation

The morphological transition of *C. albicans* (yeast-to-hyphal) represents a critical stage in biofilm formation and is the primary mechanism for cellular invasion. Inhibiting hyphal formation effectively prevents cellular infection. Figure 5C investigates the effects of TTO and its three small-molecule derivatives on *C. albicans*’s morphological transition. In the KB group, *C. albicans* exhibited active morphological transition, with distinct hyphal structures and attached yeast cells observed. In the TTO group, the proportion of yeast cells was higher than the control but lower than the 4SYC group, with a small amount of short hyphae present; cells in the 4SYC group were uniformly distributed in round or oval shapes, showing almost no hyphal structures; in the TPYX and γSYX groups, hyphal length and branching were reduced compared to the KB group, yet hyphae remained more pronounced than in the TTO and 4SYC groups. This study demonstrates that TTO and its three small-molecule derivatives exert antifungal effects by inhibiting *C. albicans* morphotransformation, with the 4SYC group exhibiting the most pronounced efficacy. Correspondingly, SEM analysis in Figure 2D revealed that 4SYC caused the most severe disruption to the membrane surface of *C. albicans* hyphal cells, consistent with the findings of this study.

## 4. Discussion

Research findings indicate that the primary components of Tea tree oil are 4-terpineol at 26.54%, γ-terpinene at 20.14%, and terpinene at 17.26%. The overall composition of TTO is largely comparable to other TTOs, though some differences exist. This variation may stem from variations in extraction techniques or discrepancies in analytical instruments and methodologies [19]. Overall, these results align with the typical chemical profile of TTO [20]. While TTO is generally considered safe for topical use, we did not directly assess its cytotoxicity toward host cells at the antifungal concentration used. Based on experiments examining the effects of various TTO on the growth curves of *Candida albicans*, we found that a concentration of 2 μL/mL of TTO can inhibit the growth of *C. albicans*; however, due to practical constraints, this study did not compare TTO with its major components.

We acknowledge that a positive control (e.g., amphotericin B) was not included in this study, as the primary focus was on elucidating the ferroptosis mechanism rather than benchmarking antifungal efficacy. The negative control for bactericidal efficacy is not shown in Figure 2A, but it was used to calculate the bactericidal rate (Figure 2B). Experiments on the fungicidal efficacy of TTO at different concentrations and its three small-molecule compounds against *C. albicans* demonstrated that the 4SYC group exhibited the strongest fungicidal effect against *C. albicans*. Furthermore, 4SYC played a primary role in TTO’s killing action against *C. albicans*. This indicates that 4-terpineol is the primary antifungal and bioactive component in TTO, consistent with findings by Mertas et al. [19]. SEM images of *C. albicans* in both yeast and hyphal stages after treatment with TTO and its major small-molecule compounds revealed that TTO and its small-molecule compounds significantly inhibited the invasiveness of *C. albicans* hyphae by disrupting membrane structures. With 4SYC exhibiting stronger disruptive effects, indicating its primary role in disrupting *C. albicans* hyphal morphology within TTO. Rong Liu et al. also observed significantly higher effects in the 4SYC group compared to other treatments when examining the impact of TTO and its major components on *Sclerotium rolfsii* mycelium and spores via SEM [7]. This likely indicates that TTO and its major compounds exhibit comparable antifungal efficacy and membrane disruption potential against different fungi, with 4SYC playing the primary role in inhibition. Furthermore, Rong Liu et al. observed that α-terpineol also effectively disrupts cell membranes, suggesting that, under structurally similar conditions, alcohol compounds may exhibit stronger membrane disruption effects than alkenes [7].

Regarding the transcriptomics of hyphal formation, we identified a total of 423 significantly differentially expressed genes, including 175 up-regulated and 248 down-regulated genes. Based on the results of the GO enrichment analysis, TTO treatment primarily inhibits the formation and maintenance of the hyphal state in *C. albicans* by inducing oxidative stress, disrupting iron homeostasis, compromising cell wall integrity, and affecting ergosterol metabolism. Notably, there was a significant enrichment of functions related to oxidoreductase activity and iron ion binding, providing transcriptomic evidence for the mechanism of TTO-induced ferroptosis that we previously proposed [21]. Furthermore, KEGG enrichment analysis reveals that TTO treatment systematically disrupts the metabolic network of *C. albicans* hyphae, affecting pathways such as glutathione metabolism (the core pathway of ferroptosis), sterol biosynthesis (related to cell membrane integrity), ABC transporters (associated with drug resistance), and several energy and amino acid metabolism pathways. The changes in the glutathione metabolism pathway are particularly pronounced, providing key pathway-level evidence for the mechanism by which TTO induces ferroptosis. Overall, the results from differential gene expression analysis, GO enrichment, and KEGG enrichment corroborate one another, collectively forming a multidimensional molecular evidence chain for the antifungal mechanism of TTO. We acknowledge that this study did not examine metabolic pathway alterations across different morphological forms of *C. albicans* (yeast vs. hyphal). Given that metabolic activities are known to differ between these morphologies, future studies should investigate whether the observed pathway changes are morphology-dependent.

Based on transcriptomic analysis, a study was conducted to investigate the effects of TTO on ferroptosis and pathogenicity in *C. albicans*. Results from ferroptosis-related experiments revealed that terpinene significantly induced ROS accumulation and increased lipid peroxidation in *C. albicans*, and that treatment with a ferroptosis inhibitor enhanced the viability of *C. albicans* cells following TTO exposure. In experiments on the pathogenicity of *C. albicans*, it was found that TTO can inhibit pathogenicity by reducing adhesion, suppressing hyphal transformation, and inhibiting biofilm formation, with 4-terpineol playing a primary role and demonstrating greater efficacy than TTO. Shen et al. first demonstrated ferroptosis during the development of conidia in Magnaporthe oryzae (rice blast fungus), revealing a mechanism by which it influences pathogen virulence through the regulation of iron metabolism dynamics [22]. This suggests a potential link between ferroptosis and pathogenicity in *C. albicans*. Meanwhile, the plant-derived compound naringin possesses antioxidant activity and can interfere with the spore cell death/ferroptosis process—a process critical to the pathogenicity of Mycobacterium oryzae—making it a potential novel antifungal agrochemical [23]. This suggests that TTO may regulate the pathogenicity of *C. albicans* through ferroptosis. Whether the ferroptosis-mediated mechanism observed in *C. albicans* applies to other fungal species with lower TTO sensitivity remains to be determined, and future comparative studies are needed.

## 5. Conclusions

Through transcriptomic analysis and a series of in vitro experiments, this study systematically investigated the mechanisms underlying the antifungal activity of tea tree oil and its major small molecules against *Candida albicans*. The main conclusions are as follows: First, the major components of TTO are 4-terpineol at 26.54%, γ-terpinene at 20.14%, and terpinene at 17.26%. Second, this study is the first to demonstrate that TTO can inhibit the pathogenicity of *C. albicans* through ferroptosis. Transcriptomic analysis revealed that TTO treatment disrupted iron metabolism in the fungi and caused significant differences in the glutathione metabolic pathway. Further experiments demonstrated that the treated group exhibited characteristic features of ferroptosis, such as ROS accumulation and increased peroxides. Additionally, the activity of *C. albicans* was significantly enhanced after pretreatment with ferroptosis inhibitors, with the small-molecule compound TPYX showing the most pronounced effect in inducing ferroptosis in *C. albicans*. Furthermore, regarding *C. albicans*, TTO and its small-molecule compounds can effectively inhibit biofilm formation by reducing adhesion and suppressing the transition to the hyphal state, and they also exhibit a certain degree of clearance activity against mature biofilms, with 4-SYC demonstrating the best efficacy.

## Figures and Tables

**Table 1:** GC-MS Data Analysis of TTO.

Serial Number	Retention Time (min)	CAS Number	English Name	Peak Area	Relative Content (%)	Match Rate (%)
1	25.2442	562-74-3	terpinenol-4	24.0110	26.54	94
2	15.7194	99-85-4	γ-Terpinen	18.2255	20.14	95
3	16.6673	586-62-9	Terpinolen	15.6261	17.26	97
4	24.5023	25246-27-9	Alloaromadendren	3.6831	4.07	96
5	14.5819	3387-41-5	Sabinen	3.6363	4.02	87
6	26.7642	98-55-5	alpha-Terpineol	3.6224	4.00	90
7	27.7578	483-76-1	δ-Cadinene	3.1123	3.44	95
8	9.3423	7785-70-8	(+)-α-Pinene	3.0387	3.36	96
9	16.3274	527-84-4	Cymene	2.8894	3.19	97
10	26.8786	21747-46-6	Leden	2.5866	2.86	97

## Data Availability

The original contributions presented in this study are included in the article. Further inquiries can be directed to the corresponding authors.

## Abbreviations

The following abbreviations are used in this manuscript:

FBS	Fetal bovine serum
MDA	Malondialdehyde
ROS	Reactive oxygen species
TPYX	Terpinene
TTO	Tea tree oil
4SYC	Terpinene-4-ol
γSYX	γ-pinene
